# *In vitro *culture and somatic cell nuclear transfer affect imprinting of SNRPN gene in pre- and post-implantation stages of development in cattle

**DOI:** 10.1186/1471-213X-9-9

**Published:** 2009-02-06

**Authors:** Joao Suzuki, Jacinthe Therrien, France Filion, Rejean Lefebvre, Alan K Goff, Lawrence C Smith

**Affiliations:** 1 Centre de recherche en reproduction animale (CRRA), Faculty of veterinary medicine, University of Montreal, Saint-Hyacinthe, QC J2S 7C6, Canada

## Abstract

**Background:**

Embryo *in vitro *manipulations during early development are thought to increase mortality by altering the epigenetic regulation of some imprinted genes. Using a bovine interspecies model with a single nucleotide polymorphism, we assessed the imprinting status of the small nuclear ribonucleoprotein polypeptide N (SNRPN) gene in bovine embryos produced by artificial insemination (AI), *in vitro *culture (IVF) and somatic cell nuclear transfer (SCNT) and correlated allelic expression with the DNA methylation patterns of a differentially methylated region (DMR) located on the SNRPN promoter.

**Results:**

In the AI group, SNRPN maternal expression is silenced at day 17 and 40 of development and a third of the alleles analyzed are methylated in the DMR. In the IVF group, maternal transcripts were identified at day 17 but methylation levels were similar to the AI group. However, day-40 fetuses in the IVF group showed significantly less methylation when compared to the AI group and SNRPN expression was mostly paternal in all fetal tissues studied, except in placenta. Finally, the SCNT group presented severe loss of DMR methylation in both day-17 embryos and 40 fetuses and biallelic expression was observed in all stages and tissues analyzed.

**Conclusion:**

Together these results suggest that artificial reproductive techniques, such as prolonged *in vitro *culture and SCNT, lead to abnormal reprogramming of imprinting of SNRPN gene by altering methylation levels at this locus.

## Background

The procedure of SCNT in mammals results in pregnancy rates much lower than those obtained *in vivo *after insemination and from transfer of embryos derived *in vitro *[1-4]. Furthermore, cloned fetuses that survive to term often have disorders such as oversized organs, increased or decreased overall growth, respiratory failure and limb malformations. In cattle and other ruminants, these abnormal phenotypes are known as the large offspring syndrome, or LOS [5,6]. Detailed examination of the extra embryonic membranes of SCNT pregnancies often highlights numerous placental abnormalities, including a reduction in the number of cotyledons, and a decrease in chorio-allantoic blood vessels. These observations are also consistent with other reports where placentomes were absent in the placenta in pregnancies that were lost between days 30 and 60 of gestation [7,8]. Together, these results suggest that improper development of the placenta may play a major role in the fetal abnormalities and low pregnancy rates in cattle SCNT. It has been suggested that the pathological phenotypes in the placental and fetal development of clones are associated with abnormal reprogramming by the host ooplasm of the donor cell used for nuclear transfer [9]. These abnormalities often disturb the epigenetic regulation mechanisms inherited from the differentiated donor cell, by altering the dynamic nature of DNA methylation and chromatin modification patterns during embryo development [10].

One of the most studied epigenetic modifications is DNA methylation of cytosine residues within CpG dinucleotides; these are often associated with transcriptional repression and implicated in maintaining genomic stability, as well as silencing repetitive elements. DNA methylation is also implicated in the regulation of genomic imprinting, genes that are exclusively expressed from only one parental allele [10]. To date, only a few imprinted genes have been characterized in cattle [11-15] and most play essential roles in fetal development and placental function. The bicistronic gene SNURF-SNRPN, referred here as SNRPN, has been extensively studied in mice and humans due to the correlation between disorders within the SNRPN differentially methylated region (DMR) and the pathogenesis of neurodevelopmental disorders known as Prader-Willi Angelman syndrome. Interestingly, decreased levels of the maternal allele methylation in the SNRPN DMR has been observed in children conceived by assisted reproductive technologies (ART), suggesting that the SNRPN methylation pattern is affected by *in vitro *culture systems [16,17]. As demonstrated previously in cattle [18], the SNRPN gene is also maternally imprinted in pre-implantation bovine embryos, with a characterized DMR. However, little is known about the effect of altered DNA methylation patterns on allelic expression of the SNRPN gene. A bovine interspecies model [*Bos indicus *(paternal genome) × *Bos taurus *(maternal genome)] that is widely used in warm climate animal breeding practices was applied to assess genomic imprinting through parental-specific polymorphisms [19,20]. Our objective here was to characterize the imprinted status of SNRPN before (day 17) and after (day 40) implantation, to determine whether the pattern of gene expression is associated with DNA methylation levels, and finally to examine short and mid term effects of *in vitro *culture on imprinting status of SNRPN gene in embryos produced by IVF and SCNT.

In this study, we show that SNRPN is maternally imprinted in pre- and post-implantation *in vivo *development. Moreover, *in vitro *culture and somatic cell cloning lead to decreased methylation of the DMR and consequently biallelic expression of the SNRPN gene.

## Results

### Development during early gestation

Table 1 shows the results of blastocyst development, day-17 and day-40 recoveries for all experimental groups. To obtain samples that were not exposed to *in vitro *culture conditions (control groups), we artificially inseminated (AI) a superovulated heifer to harvest 3 intact elongated-stage embryos at 17 d after insemination (only whole embryos were used); 4 other heifers were inseminated to recover day-40 *in vivo *fetuses (3) and their respective placentas. The *in vitro *development of IVF and SCNT embryos was assessed at day 8 of *in vitro *culture and showed that development rate to the blastocyst stage was similar for both groups. These results indicate that the prolonged handling of the oocytes during SCNT was not detrimental to the early stages of development *in vitro*, and that the SCNT protocol used did not disturb early development. To examine the development *in vivo *of *in vitro*-derived SCNT and IVF blastocysts, during the first week after transfer, groups of 10 blastocysts were transferred non-surgically to synchronous recipients and recovered at day 17 after estrus. Similar percentages of day-17 embryos were collected from the SCNT and IVF groups (P > 0.05), indicating that pre-implantation development was not affected by cloning. Nonetheless, SCNT embryos were smaller, e.g. less elongated, than their IVF counterparts, indicating that the proliferation of the trophoblasts was somewhat retarded in this group. To obtain day-40 fetuses in the IVF and SCNT groups, 1–2 embryos were transferred per recipient and viability was checked the day before slaughter. As expected survival rates at day 40 were slightly higher in the IVF group than in the SCNT group, indicating that cloned embryos are less able to sustain development beyond implantation.

**Table 1:** Survival rates of bovine oocytes subjected to AI, IVF and SCNT at pre- and postimplantation stages of development.

		Embryos recovered from uterus	
		
Group	Blastocyst development to day 7.5	day 17*	day 40*
AI	-	3	3 (75%)**
IVF	27 (34%)	5 (50%)	3 (50%)
SCNT	26 (28%)	6 (45%)	3 (30%)

* percentage of embryos were calculated from the total number of transferred embryos.

** percentage of pregnancy was calculated from the total number of AI.

### Identification of a polymorphism for allele-specific transcript analysis

**Figure 1:**
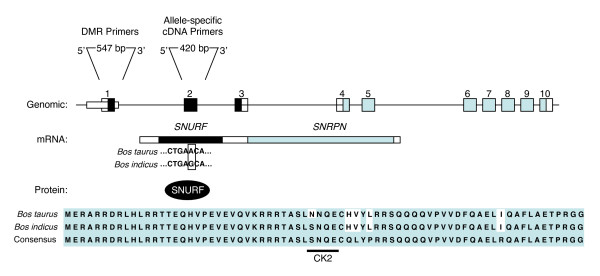
**Identification of an allele-specific single nucleotide polymorphism (SNP) on SNRPN exon 2**. Schematic representation of the bicistronic SNURF-SNRPN locus in *Bos indicus *and *Bos taurus*, depicting the progression from DNA to protein. Genomic: Numbered boxes represent exons respective to *SNURF *(black) and *SNRPN *(gray); hatched box represents the DMR analyzed. Primer positions and amplicon size for DMR and allele-specific cDNA are indicated above. mRNA: Single transcript with the SNURF (black) and SNRPN (gray) open reading frames. *Bos taurus *and *Bos indicus *cDNA sequences are aligned to indicate the SNP (G to A transition; open box) at position +151 (GeneBank AF1010140). Protein: Amino acid alignment of SNURF protein is shown for *Bos taurus*, *Bos indicus *and compared to a consensus sequence. Differences from the consensus are indicated in white boxes. The *Bos indicus *SNP leads to a change from an aspergine to a serine in the phosphorylation site for casein kinase II (CK2) (adapted from [19]).

DNA and RNA were extracted, purified and used as a template for sequencing and searching for single nucleotide polymorphisms (SNP) between the *Bos taurus *and *Bos indicus *subspecies. In comparison to the *Bos taurus *published sequence (GeneBank: AF101040), we detected an adenine (A) to guanine (G) transition in *Bos indicus *DNA located in exon 2 (position 151) of the SNURF-SNRPN upstream reading frame protein [21], the 71 amino acid protein SNURF that encompasses exons 1–3 (Figure 1). Interestingly, this *Bos indicus-*specific missence mutation leads to the substitution of an asparagine to a serine in codon 50, a position within a phosphorylation site for casein kinase II (CK2). In contrast to the widespread conservation of SNURF, *Bos taurus *SNURF differed at this position whereas our findings show that the *Bos indicus *SNURF CK2 site is homologous to other mammals, including humans [21]. Genomic DNA samples were obtained from *Bos taurus *(maternal), *Bos indicus *(paternal) and crossbred tissues (*Bos indicus *vs. *Bos taurus*). Whereas the G and A where consistently found in *Bos indicus *and *Bos taurus *samples, an overlap of G/A nucleotides was detected in the sequence chromatograms of F1 genomic DNA confirming the use of interspecies F1 crosses for allele-specific gene expression analysis. Between 15 and 20 clones were selected and sequenced for allele expression analysis of each sample.

### Allelic expression profiles of SNRPN gene at day 17

**Figure 2:**
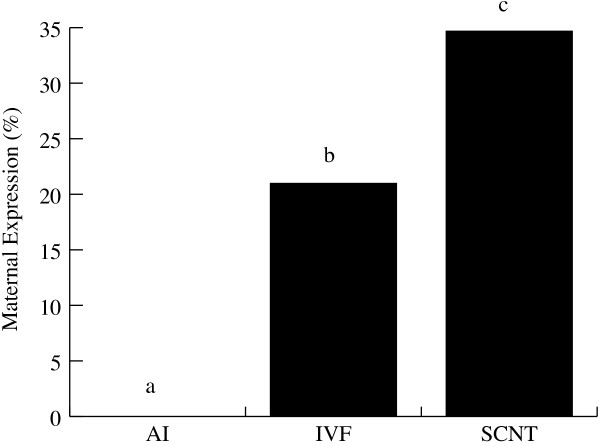
**Maternal expression analysis of SNRPN gene at day 17**. Average percentages of maternal expression in embryos produced by AI (n = 3), IVF (n = 5) and SCNT (n = 6). SNRPN reverse transcription-polymerase chain reaction fragments were cloned into a plasmid vector and sequenced for the parental SNP. Ratios were based on the total number of paternal alleles (G; *Bos indicus *SNP) found over the total number individual clones sequenced. *Subscripts represent significant differences within groups (P < 0.05).

In the AI group, expression of the SNRPN gene was exclusively paternal (Figure 2), thus demonstrating the imprinted status of SNRPN gene locus during this pre-implantation stage of development. This result also demonstrates that the *in vivo *control group showed the same SNRPN imprinting status observed in mice and humans, thus validating our bovine hybrid model. However, in the IVF group, bi-allelic expression was observed at day 17 (21% of maternal SNRPN expression), indicating that the *in vitro *culture conditions employed to produce day-8 blastocysts altered the imprinted status of SNRPN. Moreover, average levels of maternal expression were higher in SCNT than in IVF (34% vs. 21% P < 0.05) and individual SCNT maternal expression ranged between 20% and 50% (Table 2), significantly above the average IVF value. These results indicate that the protocol of SCNT alters the imprinted status of SNRPN beyond the effects of *in vitro *culture at this early stage of development and that the levels of allelic expression vary significantly between different SCNT embryos.

**Table 2:** Percentage of paternal expression of SNRPN gene and methylated CpGs islands on SNRPN DMR of AI, IVF and SCNT day-17 embryos.

	**AI**			**IVF**					**SCNT**					
			
Embryos	1	2	3	1	2	3	4	5	1	2	3	4	5	6
Paternal Expression	100	100	100	85	85	85	80	80	60	70	50	70	80	80
DMR Methylation	27.2	32.2	55.0	29.1	22.0	47.1	17.0	15.3	0.9	0.5	4.0	0.4	0.1	3.8

### Allelic expression profiles of SNRPN gene at day 40

**Figure 3:**
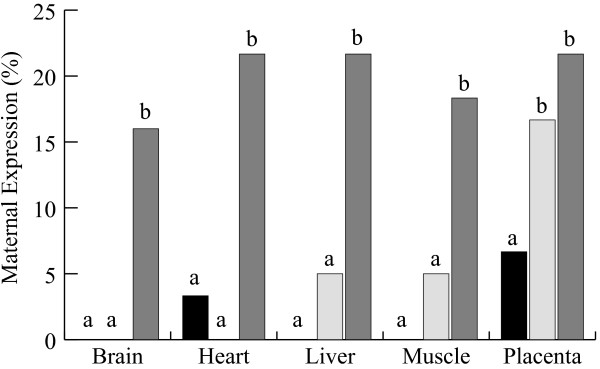
**Maternal expression analysis of SNRPN gene at day40**. Average percentages of maternal expression in tissues (placenta and fetus tissues: brain, heart, liver and muscle) obtained from embryos produced by AI (black bar) and IVF (open bars) and SCNT (gray bars). SNRPN reverse transcription-polymerase chain reaction fragments were cloned into a plasmid vector and sequenced for the parental SNP. Ratios were based on the total number of paternal alleles (G; *Bos indicus *SNP) found over the total number individual clones sequenced. a, b subscripts represent significant differences within tissues (P < 0.05).

Allele-specific transcript analysis was also performed at day 40 of development in samples of liver, muscle, brain, heart and placenta (Figure 3). All AI samples showed mono-allelic expression, i.e. exclusively paternal transcripts, except heart (3%) and placenta (7%), where low levels of maternal expression were observed. We defined samples having 10% or less expression from the normally silent maternal allele as being "leaky" but not enough to be considered bi-allelic, demonstrating that SNRPN imprinting is maintained after implantation in cattle fetal tissues and placenta. IVF embryonic tissues in general showed mostly paternal expression of the SNRPN gene. Leaky maternal expression was observed in the IVF group in liver (5%) and muscle (5%). Interestingly, bi-allelic expression was found in placenta (17%), suggesting that imprinting was not properly reestablished after *in vitro *culture, particularly in this tissue. In the SCNT group all tissues showed bi-allelic expression and maternal expression levels were higher than 15%. Heart, liver and placenta were the most affected, where more than 20% was maternally expressed. Together, results of allelic expression indicate that the SNRPN gene is maternally imprinted at pre-implantation stages and this status is maintained throughout development until day 40 in all embryonic tissues analyzed in our control group (AI). However, the placenta seemed to be affected by *in vitro *culture, since bi-allelic expression mode continued even after implantation in the IVF group and, in SCNT embryos, all tissues showed bi-allelic expression of SNRPN.

### Methylation analysis of the SNRPN DMR at day 17

**Figure 4:**
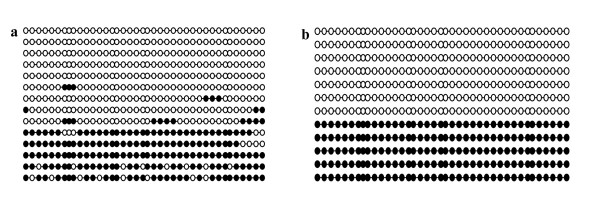
**SNRPN DMR methylation in control samples**. DNA bisulfite analysis showing the CpG methylation patterns in the SNRPN DMR of **(a) **control fibroblast donor cell derived from a day-60 *in vivo *produced fetus and **(b) **a 1:1 mixture of paternal (unmethylated)/maternal (methylated) DNA recovered from sperm and oocytes, respectively. Each line represents an individual clone that was sequenced. Black filled circles represent methylated CpG islands, and open circles indicate unmethylated CpG sites.

Once imprinting status was characterized, we assessed the methylation of the SNRPN DMR. Genomic DNA was extracted and, after bisulfite reaction, the ratio of methylated CpG sites over the 39 CpG sites present in the DMR. Previous studies had shown that, in day-17 preimplantation embryos, parent of origin methylation was represented by roughly 40 to 50% of methylated versus unmethylated sites [18]. To validate the method used for DNA methylation analysis, we mixed equal proportions (50% of each) of bisulfite treated DNA extracted from germinal vesicle (GV) oocytes (fully methylated CpG sites) with sperm (unmethylated CpG sites). For all samples analyzed, between 15 and 20 clones were selected and sequenced for CpG content. Figure 4 shows representative CpG methylation analysis at the SNRPN DMR obtained from the bisulfite treatment. The percentage of paternal alleles was approximately 38% in somatic cells and our standard 1:1 control, indicating that our analysis represented an unbiased contribution from each parental allele. These results were also consistent with the methylation levels found in previous studies [18].

**Figure 5:**
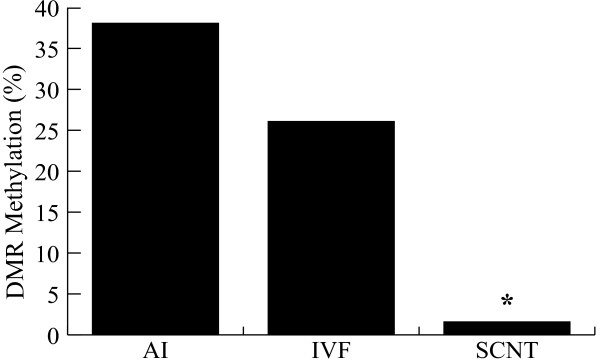
**SNRPN DNA methylation patterns at day 17**. Average percentage methylation levels of day-17 embryos derived from AI, IVF and SCNT. Bisulfite methylation data was obtained by computing the number of methylated CpG sites over the total number of CpG sites. *Subscripts represent significant differences within groups (P < 0.05).

Once the method of SNRPN DMR analysis was validated we assessed the methylation levels in the day-17 AI control embryos. Individual patterns of methylation were very similar to previous reports [18]. We confirmed that AI day-17 embryos maintained differentiated methylation patterns inherited from gametes, as roughly 40% of the SNRPN DMR was methylated (Figure 5). Methylation ratio in the IVF group was lower but not significantly different from the AI group (38% vs. 26%, P > 0.05; Figure 5), indicating that *in vitro *culture effects were only slightly detrimental to methylation maintenance. In contrast, severe loss of methylation was observed in the SCNT group, where less than 2% of methylated sites were observed. Similar results were found in clones produced by traditional methods of SCNT using zona-intact enucleation [18] confirming that the hand made cloning (HMC) method for SCNT [22] results in a similar methylation outcome. Interestingly, embryos with lower DMR methylation levels also showed higher levels of bi-allelic expression, particularly in the SCNT group (Table 2).

### Methylation analysis of the SNRPN DMR at day 40

**Figure 6:**
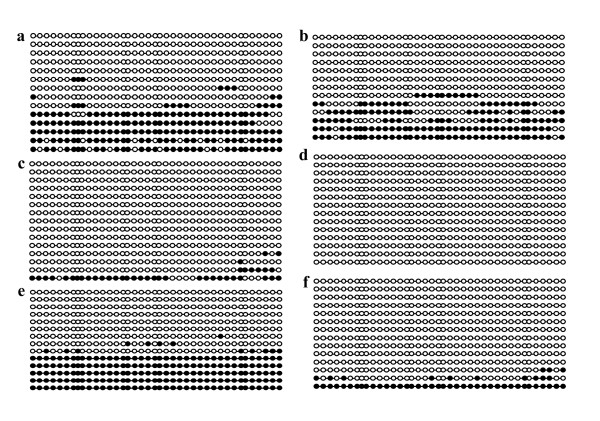
**SNRPN DMR methylation in day-40 tissues**. Representative samples of bisulfite methylation analysis of placenta (left column) and brain (right column) of AI **(a, b) **IVF **(c, d) **and SCNT (**e, f**) samples. Each line represents an individual clone that was sequenced. Black filled circles represent methylated CpG islands, and open circles indicate unmethylated CpG sites.

**Figure 7:**
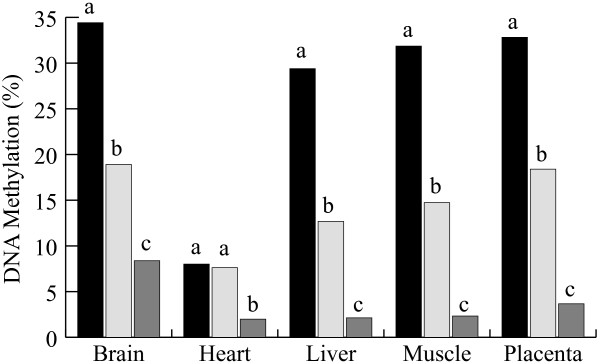
**SNRPN DNA methylation patterns at day 40**. Average percentage methylation levels in tissues (placenta and fetus tissues: brain, heart, liver and muscle) obtained from embryos produced by AI (black bars) and IVF (open bars) and SCNT (gray bars). Bisulfite methylation data was obtained by computing the number of methylated CpG sites over the total number of CpG sites. *Subscripts represent significant differences within groups (P < 0.05).

Similar methylation patterns to those seen in AI day-17 embryos were also observed in AI day-40 fetuses (Figure 6). Almost all tissues showed 40% of overall methylation, supporting the hypothesis that parent of origin methylation is maintained throughout embryo development. Surprisingly, heart samples showed very low levels of methylation in all day-40 fetuses analyzed (Table 3) even though gene expression was mostly mono-allelic in this tissue. In the IVF group, significantly lower methylation levels were found in all tissues particularly heart, where methylation ratios were comparable to the AI control (Figure 7). As at day 17, SCNT day-40 embryos showed loss of methylation levels in every fetal tissue and the placenta, indicating that abnormal methylation levels were maintained throughout early gestation and suggesting that methylation failures acquired during early stages persist throughout development.

**Table 3:** Percentage of paternal expression of SNRPN gene and methylated CpGs islands on SNRPN DMR of AI, IVF and SCNT tissues of day-40 fetuses and placenta.

		Paternal Expression (%)					Methylation (%)				
		
Groups	fetuses	liver	muscle	heart	brain	placenta	liver	muscle	heart	brain	placenta
AI	1	100	100	90	100	100	25.3	35.2	8.1	33.7	31.6
	2	100	100	100	100	80	33.0	33.2	7.6	30.2	34.7
	3	100	100	100	100	100	29.7	27.5	8.4	39.6	30.6

IVF	1	90	90	100	90	100	3.5	10.3	3.8	21.1	20.4
	2	95	95	100	80	70	14.3	9.8	8.8	8.1	14.5
	3	100	100	100	100	80	20.9	15.4	10.6	26.5	20.6

SCNT	1	75	70	90	80	80	1.6	4.0	1.2	7.6	2.0
	2	100	90	70	90	80	3.4	1.4	1.3	10.9	8.6
	3	70	85	75	80	75	3.4	1.8	3.4	6.5	1.0

### Association between SNRPN allelic expression and the DMR methylation

**Figure 8:**
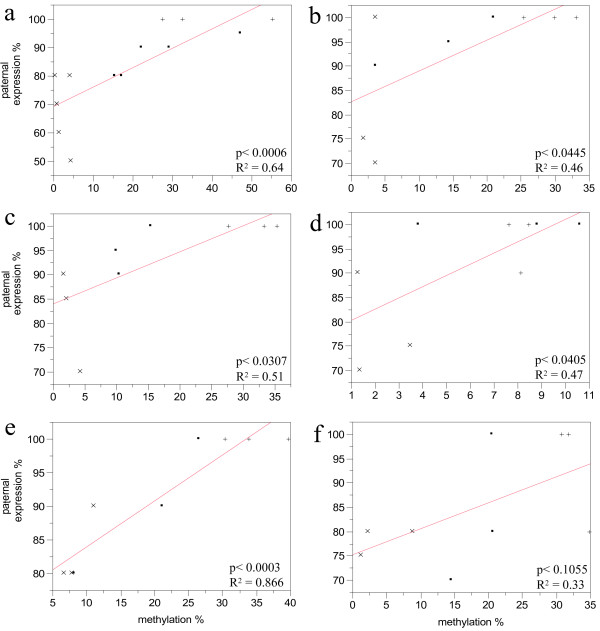
Bivariate analysis of SNRPN DMR methylation and paternal expression values. **a) **day-17 embryos, **b) **day-40 liver, **c) **muscle, **d) **heart, **e) **brain and **f) **placenta. **(.) **IVF, **(+) ***in vivo*, **(x) **SCNT.

Interestingly, when results from allelic expression and methylation ratio were combined, particular patterns were observed in different tissues (Table 3). In an attempt to correlate methylation patterns with expression, a bivariate analysis was performed on data from day-17 embryos and from each tissue from day-40 fetuses (Figure 8). A highly significant positive correlation was found between expression and methylation in day-17 embryos (P < 0.01), and brain of day-40 fetuses (P < 0.01). Day-40 liver, muscle and heart also showed a significant but less tight correlation between expression and DMR methylation (P < 0.05). Interestingly, placenta tissues showed the lowest correlation between expression and methylation patterns (R^2 ^= 0.3; P > 0.1), supporting the notion that methylation does not play a critical role in imprinting gene expression in this tissue [23]. Together, these results suggests that in general methylation of SNRPN DMR is positively associated with allelic expression, however the association seems to be stronger in some tissues than others.

## Discussion

The use of a *Bos indicus/Bos taurus *interspecies model enabled us to provide additional information on imprinting regulation of the SNRPN gene by analyzing simultaneously the methylation status and the allelic expression of SNRPN gene during the transitional period between the elongated pre-implantation embryo to the initial post-implantation stage in embryonic and extra-embryonic tissues in cattle. Furthermore, we characterized alterations to the transcription patterns and DMR methylation of SNRPN cause by exposing early stage embryos to *in vitro *culture conditions and SCNT, demonstrating that ART can significantly disturb the epigenetic control of imprinted genes.

Imprinted gene profiles have been previously reported in ruminants [11,13,14], and the interest in these genes arises from their implication in embryo and fetal development. In many clinical cases in humans or livestock animals, an association of abnormalities found in pregnancies resulting from ART and abnormal expression of imprinted genes is often found. A better knowledge of imprinted genes could provide clues to understand and improve *in vitro *culture conditions. Ultimately, in SCNT, imprinting analysis is essential to define the mechanisms underlying the ability of the oocyte to reprogram the epigenetic memory of somatic donor cells.

The maternally imprinted SNRPN gene has been extensively studied in mice and humans [24] due to its association to Angelman and Prader-Willi syndromes; it has now also been putatively linked to ART and infertility [16]. Although ART has been extensively used in the bovine species, little is known about SNRPN imprinting status. Although we confirm previous findings that the DMR methylation pattern in IVF embryos is not different from the AI group [18], we have found bi-allelic SNRPN expression at day 17 in preimplantation IVF embryos subjected to *in vitro *culture up to day 8. Recently, bi-allelic expression of SNRPN and a number of other imprinted genes was found in day-8 bovine blastocysts, suggesting that mono-allelic expression may not be required for most imprinted genes during preimplantation development and occur in a gene- and time-dependent manner [25]. However, results from our AI control showed no sign of bi-allelic expression, whereas the bi-allelic expression found in day-17 IVF embryos was also observed at post-implantation stages, suggesting that placenta tissues might be more susceptible to the effects of *in vitro *embryo culture. In our study, bi-allelic expression extended to post-implantation development in day-40 placenta, and since pre-implantantion embryos are mostly composed of extraembryonic tissue, this indicates that imprinting was already perturbed during earlier development. In the placenta, imprinting is probably regulated by mechanisms other than DNA methylation. In fact, studies in mice have revealed that imprinting establishment of *Xist *gene does not require the DNA maintenance methyltransferase DNMT1 [21]. Instead, the process of X chromosome inactivation is rather dependent on histone modifications associated with transcriptional repression by histone H3 methylated at lysine 9 (H3K9me) and 27 (H3K27me) as well as the Polycomb H3K27 methyltransferase complex, which is involved in the maintenance of transcriptional repression [21,24]. In support of these results we found the lowest association between methylation and allelic expression in placenta (Figure 8f). However, DMR methylation levels were diminished also in most embryonic tissues and we do not exclude the possibility of further complications due to loss of methylation. A wide range of genes can be affected by *in vitro *embryo culture, as reported for preimplantation mouse embryos [26]. Nonetheless, these results provide support for the hypothesis that placental tissue is more affected by *in vitro *culture than embryonic tissues [6,26,27], and pregnancy abnormalities could relate to problems observed later during gestation, when the placenta becomes more important to fetal development.

SNRPN DMR in SCNT embryos and fetuses showed severe loss of methylation and maternal expression of around 30% in day-17 embryos (Figure 2). Bi-allelic expression also persisted in day-40 extra-embryonic and embryonic tissues, although maternal expression was less pronounced than in day-17 embryos. It is likely, therefore, that problems with pregnancies are initially due to defects at the early stages of placenta development, which are aggravated as the embryo develops further. In support of this hypothesis, some underdeveloped blastocysts (less elongated) were found at day 17 in SCNT but not in the IVF group. However, at day 40 no pathologies were found in SCNT embryonic tissues, e.g. their sizes were normal and pregnancy progressed until collection, but their placenta had no visible placentomes and limited signs of vascularization of the choriallantoic membrane. Similar observations have been previously reported in cross-species clones by Dindot *et al *[7]. These results found in pre-and post-implantation trophoblastic tissue raise the question whether abnormal expression observed in embryonic tissues is a consequence of donor cell reprogramming failures, or if placenta malfunction would eventually account for abnormal expression of imprinted genes observed in day-40 fetal tissues. Another possibility is that defects caused by SCNT, other than those related to genomic imprinting, affect cell fate choices in early development and that cells with more anomalies (competence, cell division, polyploidy) are preferentially incorporated into the trophectoderm rather than the inner cell mass, as seen in tetraploid complementation [28]. However, loss of methylation in embryonic tissues seems to be associated with reprogramming failures of the donor cell [29]. Studies indicate that the methylation of imprinted genes is maintained throughout embryo development and determines either the repression or expression of these genes while the rest of the genome becomes demethylated [10]. Probably failures in donor cell reprogramming and detrimental effects of *in vitro *culture could together account for the severe abnormalities found in the placentas of SCNT.

To our knowledge, this is the first study to compare methylation directly with imprinting status in different tissues during pre- and post-implantation stages of development. More studies are needed to determine whether, in cattle, there are other DMRs acting on the same locus, or if another imprinting mechanisms, i.e. histone acetylation, play a role as important as DNA methylation in the control of SNRPN expression.

## Conclusion

Bi-allelic SNURF-SNRPN gene expression was found in IVF and SCNT preimplantation embryos subjected to *in vitro *culture, which persisted only in fetal tissues of cloned cattle. Loss of methylation was also found in embryonic and extra-embryonic tissues of pregnancies derived by IVF embryos cultured *in vitro*. Furthermore, bi-allelic expression was observed, in placenta, but not fetal tissues. Thus, we postulate that the detrimental effects of *in vitro *culture on pre- and post-implantation, play an important role in the establishment of SNRPN imprinting, particularly in placenta tissues and, in SCNT, is aggravated by failures in donor cell reprogramming.

## Methods

All procedures were performed in compliance with the Guide for the Care and Use of Agricultural Animals in Research and Training, approved by the animal experimentation committee of the Université de Montréal sanctioned by the Canadian Council on Animal Care.

### Nuclear donor cells

Fetal fibroblast cell cultures were established from a 60-day-old crossbred fetus produced by AI of a Holstein (*Bos taurus*) heifer with semen from a Nellore (*Bos indicus*) bull. Fetal tissues (brain, heart, liver, muscle and placenta) were minced manually and digested with 0.25% trypsin and 0.02% EDTA (Gibco BRL, Burlington, ON, Canada) at 37°C for 10 min. Isolated cells were washed and cultured for approximately 4 d in Dulbecco modified Eagle medium (DMEM; Gibco BRL) supplemented with 10% fetal bovine serum (FBS; Gibco BRL) and 0.5% antibiotics (penicillin 10000 U/ml and streptomycin 10 000 μg/ml; Gibco BRL) at 37°C in 5% CO_2_. When the cultures were confluent, primary passage cells were frozen in culture media supplemented with 10% dimethyl sulfoxide and stored in liquid nitrogen. Donor cells were thawed at 37°C for 1 min and cultured to confluence for a maximum of 5 passages before use as donor cells.

### Host oocytes

Cattle ovaries were collected from a local abattoir and transported to the laboratory in saline at 30–35°C within approximately 2 h after slaughter. Follicles with diameters between 2 and 10 mm were punctured with a 18-gauge needle, and cumulus oocyte-complexes (COC) with approximately 4 to 6 layers of cumulus cells and homogeneous oocyte cytoplasm were washed in Hepes-buffered tissue culture medium (TCM-199; Gibco BRL) supplemented with 10% (vol/vol) FBS. Groups of 20 COC were placed in 100 μl of bicarbonate-buffered TCM-199 supplemented with 10% FBS, 50 μg/ml LH (Ayerst, London, ON, Canada), 0.5 μg ml/ml FSH (Folltropin-V; Vetrepharm, St-Laurent, PQ, Canada), 1 μg ml/ml estradiol 17-β (Sigma-Aldrich, St. Louis, MO), 22 μg ml/ml pyruvate (Sigma-Aldrich), and 50 μg/ml gentamicin (Sigma-Aldrich). After 19 to 20 h of *in vitro *maturation, cumulus cells were removed from the COC by vortexing for 2 min in PBS and 0.2% hyaluronidase (Sigma-Aldrich). Only oocytes with homogeneous cytoplasm and intact cell membrane were selected for micromanipulation.

### *In vivo *and *in vitro*-derived embryos

Production of embryos and fetuses for *in vivo *and *in vitro *controls, as well as donor cells were conducted as described previously [18]. Briefly, *in vivo*-derived embryos were obtained from Holstein heifers that were superovulated by intramuscular injection of porcine FSH (Folltropin-V) given every 12 h in decreasing doses starting with 60 mg at day 9, 50 mg at day 10, 30 mg at day 11 and finally 20 mg at day 12 of the estrous cycle. At day 13 cows received an injection of 500 μg of cloprostenol (Estrumate; Schering-Plough Animal Health, Pointe-Claire, QC, Canada) and were artificially inseminated (AI) in the next 48 h after cloprostenol injection.

*In vitro*-produced embryos were derived using standard protocols of *in vitro *maturation (IVM), fertilization (IVF) and culture (IVC) [12]. Briefly, bovine ovaries were obtained from a local slaughterhouse and transported to the laboratory within 4 h in saline at 32°C. For IVM, groups of 20–25 COC were cultured in 100 μl drops of Tyrode medium supplemented with 0.6% BSA (fraction V; Sigma-Aldrich), lactate, pyruvate, gentamicin, and heparin (10 μg/ml). For IVF, frozen-thawed spermatozoa were washed and centrifuged through a Percoll (Sigma) gradient and diluted to 10^6 ^live spermatozoa/ml. After 24 of IVM, COCs were added to fertilization drops and at 20 h following the start of incubation with spermatozoa, oocytes were denuded of cumulus cells by brief shaking. For IVC, putative IVF zygotes were transferred to 25 μl drops of synthetic oviduct fluid (SOF medium) and cultured for 8 d, with additional 25 μl of SOF medium. The same IVC conditions were used for the oocytes reconstructed by SCNT.

### Nuclear transfer

The SCNT protocol used was a slight modification from a previous report of hand made cloning (HMC) [22]. Oocytes were selected in groups of 100 and placed in 1.5 mg/ml pronase in TCM 199 supplemented with FBS 10% for about 4 min. Zona-free oocytes were washed thoroughly in TCM supplemented with FBS 20% for 3 min and cultured in 0.4 μg/ml demecolcine for at least 30 min. Treated oocytes with a visible protruding membrane were placed in medium supplemented with 5.0 μg/ml cytochalasin and 10% FBS and manually bisected using a micro blade on a stereomicroscope. After bisection, oocytes were stained with 2 μg/ml Hoescht 33342 and checked for the absence of chromatin using a short exposure to UV fluorescence. Nuclear donor cells were thawed, washed and placed in 50 μl of culture media (DMEM, supplemented with 10% FBS and 0.5% antibiotics). Nuclear transfer was performed using confluent cells that were maintained in culture for 3–5 passages. Cytoplasts were placed individually in a 50 μl drop containing 500 μg/ml of phytohemagglutinin (Sigma) for about 3 sec and then quickly positioned over a single donor cell placed at the bottom of the dish. After attachment of the donor cell, the cytoplast-somatic cell couplets were placed in 0.3 M mannitol solution containing 0.1 mM MgSO_4 _and 0.05 mM CaCl_2 _and exposed to a 1.2 kV electric pulse lasting 70 μsec. After electrical stimulation, couplets were washed and cultured individually in 10 μl drops of 6-dimethylaminopurine (DMAP, Sigma-Aldrich) for 3 h. After DMAP treatment, reconstructed oocytes were washed and cultured in 40 μl drops of SOF modified medium supplemented with 0.8% BSA-V fatty acid free (Sigma-Aldrich) under equilibrated mineral oil at 39°C in a humidified atmosphere of 5% CO_2 _and 5% O_2_. Embryos were cultured in groups of 4 per drop in small individual wells (500 μm diameter) prepared with a sterile needle to avoid separation of blastomeres during development. Reconstructed embryos were cultured *in vitro *for a period of 8 d.

### Day-17 elongating embryos and day-40 fetuses

The estrous cycle of Holstein heifers was synchronized by an injection of 500 μg of the prostaglandin F2α analogue, cloprostenol (Estrumate, Schering Canada Inc). Six to 8 d after the standing heat, day-8 *in vitro*-produced or SCNT blastocysts were transferred to the uterine horn ipsilaterally to the corpus luteum. Embryos were washed with TCM-199 Hepes-buffered medium supplemented with 10% of FBS, loaded into a 250 μl straw and transferred to recipient heifers. One group of heifers received between 10 to 15 day-8 IVF or SCNT embryos and allowed to develop for another 9 d in the uterine horn. Day-17 elongated embryos were non-surgically recovered by flushing the uterus of the recipient heifers with PBS using a Foley catheter. Embryos were removed from the flushing media and inspected to select those that were recovered intact. After selection, embryos were washed three times in PBS and frozen individually at -70°C in 0.2 ml of distilled water. Only those embryos that were recovered intact were used for the experiments. The second group of heifers was allowed to continue gestation to day-40 after SCNT or IVF. Recipients carrying fetuses with a normal heartbeat were slaughtered at the local slaughterhouse and transported to the laboratory on ice within approximately 1 h postmortem. Samples from liver, muscle, heart, whole brain and placenta (intercotiledonary allantoic membrane) were collected from each viable gestation, snap-frozen in liquid nitrogen and stored at -70°C until further analysis.

### Bisulfite sequencing

DNA was isolated from day-17 embryos and day-40 tissues using Qiagen DNAeasy extraction kit, according to the manufacture's instructions. Approximately 200 ng of total genomic DNA was used for a bisulfite treatment reaction using the EZ DNA methylation kit supplied by Zymo Research^®^, according to the manufacturer's instructions. Primers specific for bisulfite-converted DNA for SNRPN were designed according to previous publication [18]. Nested PCR amplifications were necessary due to the limited amounts of DNA (approximately 200 ng) available for analysis. Primers were designed according to bisulfite standards (no CpG sites within primers and at least 2 cytosines within primer sequence to select for converted sequences). For the outside nested PCR, the primer sequences were as follows: Forward 5'GGAAAGTTTGAGGAAATTTGAATAAGG-3'; Reverse 5'-CAAATACCCCCAAAACCTAACAAAAC-3'. The primers used for the inside nested reaction were as follows: Forward 5'-TTGGGAGGTATTATTTTGGGTTGAAG-3'; Reverse 5'-AAAAAATCAATCCAACCCCAAACCTC-3'. Each 50 μl PCR reaction contained 4 μl of bisulfite-treated DNA, 1 μl of each primer (10 μM), 2.5 μl (100 μM) deoxynucleotide triphosphates (Invitrogen), 5 μl 5× PCR buffer (300 mM Tris-HCl, 7.5 mM ammonium sulfate, 12.5 mM MgCl_2_) (Invitrogen), and 1.25 U of DNA Taq polymerase (Invitrogen). First-round PCR was performed under the following conditions: 4 min at 94°C, 2 min at 55°C, and 2 min at 72°C for two cycles, followed by 35 cycles of PCR consisting of 1 min at 94°C, 2 min at 55°C, and 2 min at 72°C. For the second round of PCR, 4 μl of the first-round sample were used, and the conditions for the PCR were the same as the first-round conditions, except that the first two cycles were omitted. Fragments were resolved in 1.2% agarose gels, followed by purification using agarose purification kit from Qiagen. Purified fragments were subcloned in pGEM T easy Vector (Promega), and cell transfection protocol was performed using competent *Escherichia coli *cells. Clones containing the appropriate inserts were sequenced using an automated sequencer. Since bisulfite converts all unmethylated cytosines, whether or not they are in CpG dinucleotides, to guanines, only sequences with greater than 95% bisulfite conversion efficiency were used for analysis (i.e., to avoid false overestimation of methylated CpGs). Nucleotides mutations or any difference within the sequence (polymorphisms) between clones with similar CpG methylation profiles were verified to ensure that unique clones were represented. We examined 39 CpG sites in a 548-bp fragment of SNRPN. Absence of strain-specific single nucleotide polymorphisms prevented the parental origin of the sequenced strands from being determined.

### Allele-specific polymorphism in cDNA

RNA was extracted using the RNAeasy Extraction kit (Qiagen) following manufacturer's instructions. Reverse transcription and polymerase chain reaction (RT PCR) was performed using Omniscript RT-PCR kit (Qiagen). cDNA was used as a template for the next PCR using primers SNRPN Forward (5'-GGAGATGCGTGACGTTGTGT) and Reverse (5'-GGTGTTCCAATACTGCTTTAACC). A 50 μl reaction was performed consisting of 5 μl 10× PCR buffer (Promega), 4 μl 25 mM MgCl_2_, 1.25 μl 10 mM dNTPs, 2.5 μl 3 M forward primer, 2.5 ml 3 M reverse primer, 2 μl DNA, and 1 ml Taq (Promega). PCR reactions were performed for 35 cycles at 94°C (2 min), 94°C (30 sec), 65°C (30 sec), 72°C (35 sec), 72°C (3 min), and held at 10°C. Fragments were resolved on 1.2% agarose gels, purified and subcloned in sequencing vectors pGEM T easy Vector (Promega) and transformed in competent *Escherichia coli *cells. Sequence analysis indicated the presence of a SNP between the *Bos indicus *and *Bos taurus *genomes. Plasmids were purified, according to Qiagen's protocol, and results examined individually for the presence or absence of the paternally expressed *Bos indicus *genome (guanine) or maternally expressed *Bos taurus *genome (adenine). Results are expressed in percentages of individual cloned sequences possessing the maternal (*Bos taurus*) over the total number of clones analyzed.

### Statistical analyses

Statistical analysis was performed using the Chi-square test. For methylation analysis, data was analyzed by computing frequency of methylated sites over the number of unmethylated CpGs islands. For gene expression, data was analyzed using Bioedit software aligning program and frequency of paternal computed over maternal allele SNP. For both cases the level of significance was set at P < 0.05.

## Authors' contributions

JS carried out the allelic expression studies, IVM, IVF and SCNT, participated in data statistical analysis and drafted the manuscript. JT and FF carried out DNA methylation analysis and participated on sample collection. AKG carried out the data statistical analysis and helped to draft the manuscript. RL carried out superovulation, synchronization of recipients, embryo transfer and sample collection. LCS conceived of the study, and participated in its design and coordination and helped to draft the manuscript. All authors read and approved the final manuscript.
